# HIV evolution and diversity in ART-treated patients

**DOI:** 10.1186/s12977-018-0395-4

**Published:** 2018-01-30

**Authors:** Gert van Zyl, Michael J. Bale, Mary F. Kearney

**Affiliations:** 1 Division of Medical Virology, Stellenbosch University and NHLS Tygerberg, Cape Town, South Africa; 2 HIV Dynamic and Replication Program, Center for Cancer Research, National Cancer Institute at Frederick, 1050 Boyles Street, Building 535, Room 109, Frederick, MD 21702-1201 USA

**Keywords:** HIV diversity, Antiretroviral therapy (ART), HIV genetics, HIV replication, HIV reservoir, HIV persistence, Expanded clones

## Abstract

Characterizing HIV genetic diversity and evolution during antiretroviral therapy (ART) provides insights into the mechanisms that maintain the viral reservoir during ART. This review describes common methods used to obtain and analyze intra-patient HIV sequence data, the accumulation of diversity prior to ART and how it is affected by suppressive ART, the debate on viral replication and evolution in the presence of ART, HIV compartmentalization across various tissues, and mechanisms for the emergence of drug resistance. It also describes how CD4+ T cells that were likely infected with latent proviruses prior to initiating treatment can proliferate before and during ART, providing a renewable source of infected cells despite therapy. Some expanded cell clones carry intact and replication-competent proviruses with a small fraction of the clonal siblings being transcriptionally active and a source for residual viremia on ART. Such cells may also be the source for viral rebound after interrupting ART. The identical viral sequences observed for many years in both the plasma and infected cells of patients on long-term ART are likely due to the proliferation of infected cells both prior to and during treatment. Studies on HIV diversity may reveal targets that can be exploited in efforts to eradicate or control the infection without ART.

## Background

A signature of HIV infection is its vast genetic diversity and rapid evolution within and between infected individuals. HIV diversity results primarily from the lack of a proofreading mechanism by its reverse transcriptase (RT) enzyme that copies its RNA genome into DNA prior to integration into the host genome where it either remains latent or is expressed using the host cell machinery. HIV diversity is also influenced by a large population size and high recombination rate [1–4]. Other factors that contribute to the high genetic diversity of HIV are host APOBEC-mediated substitutions [5, 6] and changes in the population of susceptible cells over the duration of infection [7, 8] and across different anatomical compartments, such as the brain [9–11]. HIV evolution is driven, in large part, by the selection of expressed variants that carry mutations allowing escape from cell killing or virus neutralization by host immune responses [12–15]. Immune escape is also one mechanism that allows the virus to persist within the host, with another mechanism being proliferation of latently-infected cells [16, 17]. The latter mechanism is not affected by ART and is an important reservoir for the virus during suppressive treatment [18–20]. The interplay of all these factors explains why HIV sequences within an infected individual can differ by 5% or more [12, 21]. The major implications of viral diversity are the persistence of HIV despite strong immune responses, the selection of drug resistant mutations on ART, and the difficulties it imposes on the development of vaccines and curative strategies. In this review article, we will discuss some methods used to measure and view HIV diversity, the accumulation of HIV diversity in untreated individuals, the influence that ART imposes on HIV diversity, the relationship between HIV diversity and the reservoir on ART, and how HIV diversity can lead to the emergence of drug resistant variants and virologic failure.

## Methods to investigate HIV diversity in vivo

### Single-genome amplification and sequencing

The methods by which we measure and analyze intra-patient viral populations are paramount to our understanding of HIV diversity and evolution. Early studies utilized bulk PCR amplification and cloning to measure HIV diversity and to detect the emergence of drug resistance mutations [22–25]. However, a letter by Liu et al. discussed the issues with this type of sequence analysis, especially in the context of low viral burden, showing that the resampling probability is inversely proportional to sample size—i.e. viral burden—and thus, bulk PCR and cloning can give erroneous estimates of intra-patient diversity [26]. This skewed quantitation of intra-patient sequence diversity resulted in detection of only the majority variants present in the HIV population [26–30].

In 2005, Palmer et al. [30] showed that the standard genotyping methods missed drug resistance mutations including mutations that were linked on the same viral genomes. In order to better understand intra-patient HIV populations, Palmer et al. developed an approach, based on similar approaches by Simmonds et al. [31], by utilizing limiting-dilution PCR to amplify from single HIV RNA or DNA templates [30]. Single-genome amplification or single-genome sequencing (SGA and SGS respectively) has been shown to have a low error rate of 0.003%, and a very small assay recombination rate of less than one crossover event in 66,000 bp [30]. Salazar-Gonzales et al. later showed that, in a side-by-side comparison of bulk methods to SGS, that sequences derived by bulk methods had a noticeable error rate that contributed to a statistically significant difference between the two sets of paired sequences [13]. Jordan et al. further showed that neither bulk PCR/cloning nor SGS provided more bias than the other but noted that SGS could provide a deeper look at those sequences which would be missed by bulk PCR/cloning methods [27].

### Next-generation sequencing

Although SGS has become the gold-standard assay for studying HIV populations, it can only provide a limited look—without a herculean effort—at the intra-patient population. To address the issue of finding minority variants, and generating the maximum amount of data, various platforms of next-generation sequencing have been applied to HIV. High-throughput sequencing techniques have recently become popular and provide a deeper look at the HIV populations within patients and to search for variants that might be missed with lower throughput methods, such as rare drug resistance mutations. 454 pyrosequencing by Roche Diagnostics/454 Life Sciences has been the most prevalent deep sequencing method by which intra-host populations have been analyzed. It has been used to look at HIV populations with multiple alleles at single sites as well as searching for minority variants that may contribute to virological failure on ART [32–35]. However, in contrast to SGS, the requirement of a bulk PCR step in 454 and other deep sequencing methods can introduce artifactual recombination creating variants that are not present in the original population. PCR recombination rates have been reported to range from 5.4% recombinants to up to 37% recombinants [28, 36]. To combat these recombination rates, which hinder the search for linked minority mutations in HIV populations, Boltz and Rausch et al. [36] developed an ultrasensitive SGS (uSGS) assay, performed on the Illumina Miseq platform, that reduces PCR recombination to about 0.1%. uSGS works by incorporating primer-IDs onto cDNA molecules at the RT-PCR step [37] and then ligates adaptors which limits PCR bias and recombination by avoiding PCR with lengthy primers [36] used in other deep sequencing approaches. When applied to clinical samples, uSGS gave between 30- and 80-fold more sequences than standard SGS. However, in its current version, it is limited by the fragment length that can be analyzed, about 500 base pairs. Other advancements in deep sequencing approaches have allowed for the generation of whole- or near full-length genome sequences for rapid genotyping, SNP frequency calculations, and phylogenetic analyses [38–42]. In addition, more recent advances such as the Oxford Nanopore Technologies MinION and Pacific Biosciences SMRT sequencing are rapidly gaining traction as third generation technologies for HIV analyses [43].

### Analysis of intra-patient HIV sequence data

Methods used to analyze HIV sequence data are equally important to those used to generate them. Average pairwise distance (APD) is the most common sequence-based statistic used in SGS studies as it can inform estimations of the within-host genetic diversity of the HIV populations. The traditional way to visualize the diversity of HIV populations is by phylogenetic trees. The most basic approach to phylogenetic analyses of intra-patient HIV sequence data are neighbor joining methods. Neighbor joining trees generate branch lengths solely from the absolute genetic distance between sequences and (generally) make no assumptions on either a temporal structure or rates between transitions or transversions. However, maximum-likelihood methods and Bayesian methods of phylogeny, which have also been applied to intra-patient HIV sequence sets [44–47], apply evolutionary models that account for frequencies of transitions and transversions and may consider the time of sample collection in generating the trees. Using the branch lengths on trees as surrogates for evolutionary change can provide insight into the relative levels of polymorphism between sequences and into changes in the population structure over time. Studies investigating compartmentalization or divergence over time utilize different hypothesis-testing methods, such as the test for panmixia [48, 49] or the Slatkin–Maddison test [50], to show the presence, or lack thereof, of different population structures either between anatomical compartments or at different timepoints. Analyses of intra-patient HIV sequence data have led to a better understanding of HIV transmission [12, 51], the accumulation of viral diversity prior to ART initiation [4, 12, 52], the HIV population size [3, 4], the sources of persistent viremia on ART [46, 53, 54], and the mechanisms that maintain the HIV reservoir on ART [16, 17].

## HIV genetic diversity and divergence in vivo

### Accumulation of diversity in early and chronic HIV infection

HIV transmission is a relatively inefficient process with less than 1% of heterosexual exposures resulting in transmission and most associated with a single founder virus [12, 51]. During sexual transmission, mucosal infection of the new host results in a bottleneck which selects for viruses with higher overall fitness [55]. However, in men who have sex with men (MSM) or intravenous drug users (IVDU), when the exposure risk is high, selection for fit variants is less stringent. Moreover, the transmission of a first variant statistically increases the chance that another would transmit (transmissions do not follow a Poisson distribution). Thus, multiple founding viruses are not uncommon among MSM and IVDU, but their frequency varies across studies in accordance with the variable exposure risk [55–57]. Similar to heterosexual transmission, mother to child transmission is usually associated with one variant only, suggesting a stringent bottleneck [58]. Founding viruses are more likely CCR5 tropic, although, in some studies, up to 20% may be CXCR4 tropic [51, 59, 60]. As the initial infected target cells are activated CD4+ T cells, founding viruses require a high CD4 receptor density and may be underglycosylated compared to strains from chronic infection [61].

**Fig. 1:**
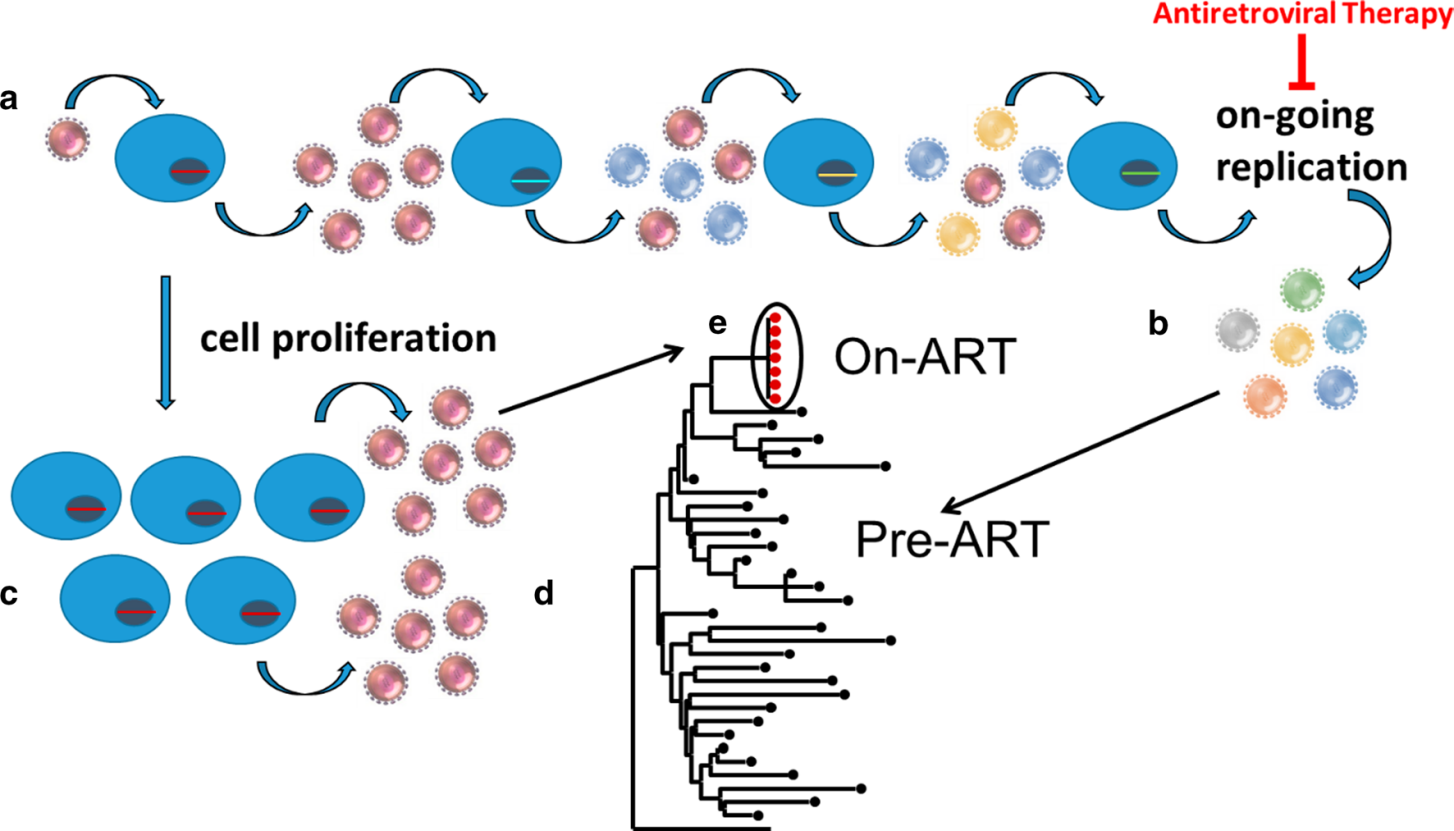
Without ART, about 10^6^–10^9^ CD4+ T cells are infected daily by HIV-1 [141] (**a**). The HIV-1 population accumulates genetic diversity with each round of viral replication at a rate of about 1 mutation in 10^5^ nucleotides copied [142] (**b**). An unknown fraction of the infected CD4+ T cells persist despite infection and undergoes cellular proliferation [16, 17] (**c**). Some clonally expanded populations of HIV-1 infected cells carry proviruses that can generate virus particles [77] (**d**). It has been shown that the identical sequences observed in persistent viremia on ART can originate from expanded clones [77] (**e**)

When only one founding virus is transmitted, the viral population is initially homogenous (Fig. 1a) but diversifies as it adapts to a new host to levels of about 1–2.5% in the viral enzymes [12] and to 5% or more in the structural genes (Fig. 1b) [12, 13, 52]. This finding was more recently demonstrated in Zanini et al. [40, 42] through whole-genome analysis of untreated patients followed longitudinally. The authors showed that the HIV genome does not evolve uniformly, with the viral enzymes having a lower rate of divergence compared to gp120 and nef. In cases with multiple founding viruses, viral populations evolve through recombination in addition to mutation [12, 56, 57, 62–64]. In non-controlling patients, HIV diversifies rapidly as variants that escape dominant cytotoxic T lymphocyte (CTL) responses are selected [12, 13, 40, 65]. However, when the HLA class I haplotype of the transmitting donor corresponds to the recipient, the transmitted variant may be a pre-adapted escape variant. Such transmission of escape variants as well as higher multiplicities of infection have been associated with a higher viral load and a more rapid disease progression in the new host [66]. In contrast, natural controllers are characterized by a greater magnitude, polyfunctionality, and breadth of CTL responses and the targeting of epitopes are conserved due to the high fitness cost of escape [67, 68]. Similar to CTL escape, escape from neutralizing antibodies through evolution of *env*, encoding the surface glycoprotein, occur as early as in the first months of infection [69]. In chronic untreated infection, viral evolution may favor the selection of strains that are less resistant to CTL killing but could infect a larger range of host cells, which may manifest as a switch from CCR5 tropic strains to dual tropic or CXCR4 tropic strains [70]. This tropism switch is associated with more rapid disease progression [71]. In untreated individuals, adaptive responses to evolving B cell epitopes and sequential antibody escape, can result in the development of broadly neutralizing antibodies. Approximately 20% of chronically infected individuals develop broadly neutralizing antibodies, usually appearing late, as they are often produced by B-cells that have evolved extensively through somatic hypermutation and B cell selection [72, 73]. As mentioned above, although HIV diversifies rapidly in patients, patients in chronic infection experience a diversification plateau independent of continued viral turnover [4].

### HIV genetic diversity on ART

The dynamics of plasma HIV RNA decay after initiating ART occurs in four phases and, oftentimes, results in an associated decline in the overall HIV genetic diversity [53, 74–76]. The first phase of decay occurs from the rapid death of most infected cells within days after initiating ART. The second phase is from the clearance of infected cells with half-lives of about 2–3 weeks. The third is from longer-lived cells with half-lives of 6-44 months and the last phase has a slope that is not significantly different from zero, likely resulting from the persistence and/or proliferation of infected cells that were previously latently-infected but, some fraction of which, produce virus upon stochastic activation [74–78]. A study by Besson et al. [79] investigated the decay of HIV DNA on ART and showed that the infected cell populations decline initially but then achieve a steady state with the persistence of about 10% of infected cells during long-term ART. The persistence of a small fraction of infected cells during ART may be achieved by maintaining a balance between cellular proliferation and cell death.

The diversity of HIV populations is influenced by the loss of the vast majority of infected cells on ART and the unveiling of identical proviruses that persist in proliferating populations of CD4+ T cells (Fig. 1c) [46, 53, 54, 80]. These monotypic sequences were first described by Bailey et al. [46] and were detected in the plasma, likely resulting from virion release from some members within clonally expanded populations (Fig. 1d, e). Maldarelli et al. [16] and Wagner et al. [17] were the first to directly show that HIV-infected cells can clonally expand and persist despite ART, and that the proviral integration site may influence this phenomenon. In one case, a provirus in an expanded cell clone was shown to match the single viral variant present at detectable levels in the persistent viremia during ART [77]. Furthermore, the virus particles produced by the clonally expanded cells were replication competent [77]. This one example is the only case, thus far, where the source of infectious virus in blood has been traced to a clone of infected cells carrying a mostly latent provirus. However, studies by Lorenzi et al. [20], Bui et al. [18], and Hosmane et al. [81] demonstrated that expanded cell clones harboring replication-competent proviruses are not uncommon among ART treated patients.

Characterizing the genetics of the HIV reservoir may help us to elucidate the mechanisms that established it prior to ART and that maintain it during ART. It is thought that the reservoir is comprised by a small number of resting, memory CD4+ T cells carrying transcriptionally silent HIV proviruses [82, 83]. Reports showing that the virus can reemerge months to years after treatment interruption in patients hoped to have been cured by bone marrow transplantation [84] or early treatment [85] support the idea that HIV can rebound from a pool of latently infected cells. However, more recent studies suggest that it may also consist of cells with transcriptionally active proviruses during ART that match those that rebound when ART is interrupted [86]. Although there is considerable patient-to-patient variation, the frequency of resting CD4+ T cells that harbor HIV proviruses detectable by PCR has been very roughly estimated to average about 1 cell in 10^3^; however, the number of latently infected cells carrying replication-competent proviruses has been reported to be much lower [5, 87]. The difference is due to the presence of a large number of defective proviruses. Ho et al. [87] described the proviruses in resting CD4+ T cells that were not induced to produce replication-competent virus after a single round of maximal T cell activation. Almost half of these proviruses had large internal deletions that preclude replication, while another third were lethally hypermutated by the host restriction factor APOBEC3G. Other defects and further analyses brought the fraction of defective proviruses up to > 98% [5]. Additionally, Ho et al. found that some of the intact proviruses were capable of producing infectious virions following a second round of activation [87], even though they had not been induced by the prior activation. Bui et al. [18] confirmed this finding and showed that sequential rounds of activation induced proliferation and expression from expanded cell clones.

Long-fragment PCR and sequencing revealed the proviral population structure in patients prior to ART and how the structure changes on long-term ART [5]. Early after infection, a large proportion of proviruses have ABOBEC-induced hypermutations and few have large internal deletions. However, as hypermutated proviruses produce and present aberrant peptides on HLA class I and are recognized by CTL, they are often eliminated whereas those with large internal deletions, and not producing antigen, may persist and continue to expand [88]. In contrast, reservoir cells harboring fully intact, replication-competent proviruses have been reported to be resistant to CTL killing, even though the viruses they release upon in vitro stimulation can be recognized by CTL [88]. This resistance to CTL killing may be due to a large fraction of the infected cells being transcriptionally silent in vivo and may explain the stability of this small pool of “true” reservoir cells [78].

### Controversy of ongoing HIV replication during ART

Residual viremia per se is not evidence for ongoing replication. Current ART inhibits attachment and fusion, reverse transcription, integration, or particle maturation after release. However, it does not prevent virus production or release which requires the transcription of provirus, translation, virus assembly and exocytosis. Considering this, as long as infected cells persist and may become activated, viral release is possible, even in the absence of the infection of new cells. Although it has been shown that one mechanism that maintains the HIV reservoir is the persistence and proliferation of cells infected before the initiation of ART [16, 17, 19, 20, 38, 39, 77], there is continued debate as to whether the reservoir can also be maintained from ongoing viral replication in potential ART sanctuary sites, such as lymph nodes (LN) [44, 89–92] with subsequent trafficking of recently infected cells into the blood [44, 93]. If ongoing replication in tissues maintains the HIV reservoir, then preventing infection of new cells by developing antiretrovirals that better penetrate sanctuary sites, such as LN, would be a high priority. Conversely, if current ART is fully effective at blocking full cycles of viral replication in both tissues and blood, then elimination of proliferating and long-lived infected cells would be the highest priority to achieve an HIV-1 cure. It is therefore critical that the efficacy of current ART be fully understood to identify the most appropriate curative strategy.

Residual viremia due to ongoing viral replication, in patients without drug resistance, would require the presence of drug sanctuaries where the drug penetration is insufficient, allowing ongoing rounds of infection. Evidence of poor drug penetration in LN and mucosa associated lymphoid tissue (MALT) exist [90] and recently an investigation using 454 sequencing and a Bayesian evolution model on samples from LN tissue and blood of 3 patients reported evidence of evolution in LN with trafficking to the blood [44]. The authors concluded that the reservoir is replenished by ongoing replication and suggest the need for better ART with improved penetration into drug sanctuaries. These findings have, however, not been reproduced by other investigators or by applying different models of evolution on the same dataset [94]. If ongoing replication is important in replenishing the reservoir, viral diversification would continue in most patients on therapy and newly emergent variants would be detectable in the periphery as infected cells migrate between compartments. However, most studies of patients on long-term suppressive antiretroviral regimens have not found evidence of sequence diversification from pre-therapy in blood or tissues [41, 45, 46, 53, 54, 95]. Also, if low level viremia was due to ongoing HIV replication as a result of inadequate suppression of replication by triple combination therapy, the addition of a fourth drug, referred to as therapy intensification, would result in a decreased viral load. However, most investigations reported no viral load reduction with treatment intensification [96–99]. Taken together there exists no conclusive evidence that modern combination ART is inadequate and contributes to viral persistence in individuals with viral loads below the detection limit of commercial assays.

Most studies addressing the question of ongoing replication on ART analyzed HIV sequence data in longitudinal samples for evidence of evolution of virion RNA or proviral DNA in adults who initiated ART in chronic infection [44, 46, 53, 54, 86, 100], in adults who initiated ART in early infection [53, 54], and in perinatally-infected infants [101, 102]. Performing SGS on individuals in early infection makes it easy to detect the mutations that accumulate with viral replication since the background genetic diversity is typically low. Using measures of diversity, divergence, and increasing branch lengths on phylogenetic trees over time, significant changes in HIV populations have not been reported in patients with sustained suppression of viremia on ART [53, 54, 102, 103] and suggest that the HIV reservoir is likely maintained largely, if not solely, by the persistence and expansion of cells that were infected prior to the initiation of treatment. However, most studies looking for evidence of HIV evolution on ART due to viral replication have been conducted on blood samples. Fewer studies have been performed on tissues collected from various anatomical sites. Results of studies on HIV evolution during ART in tissues, including those using nonhuman primate models, have been conflicting with some showing evidence of viral compartmentalization and evolution [44] while others claim the opposite conclusion [104]. The conflicting outcomes may result from differences in the methods used to perform the sequencing (deep sequencing vs. SGS), from the methods used to analyze the data (neighbor joining vs. Bayesian phylogenetics), whether the identical variants are collapsed to a single sequence or not [105], or simply from sampling error. It is obvious that more studies are needed to determine if ongoing cycles of HIV replication occur in any tissues during ART to levels that could sustain the reservoir and lead to viral rebound when ART is interrupted.

### HIV compartmentalization

Viral compartmentalization describes tissues or cell types where viral replication occurred but anatomical barriers restrict both ingoing and outgoing viral gene flow [106]. As discussed earlier, one theory is that the viral reservoir is maintained by ongoing HIV replication in sanctuary sites where drug penetration is sub-optimal [90]. In addition to the LN, the gut lymphoid tissue has also been posited as another such site of compartmentalization. A study by van Marle et al. [107] analyzed samples from the esophagus, stomach, duodenum, and colorectum and found evidence of compartmentalization in the *nef* region of the HIV genome. Furthermore, a study by Yukl et al. [108] showed that the overall burden of HIV within the gut is much higher than in the blood which may suggest that ongoing replication during ART persists within this compartment. Along these lines, a later study by Rueda et al. [109] showed increased and prolonged activation of the immune system within the gut, suggesting that immune cells were being exposed to viral protein. In contrast, Imamichi et al. showed a lack of compartmentalization between the proviral sequences derived from PBMC and from the ileum and colon [110]. This result was later corroborated by Evering et al. [45] who showed no difference in proviral sequences from the blood or gut mucosa. Evering further demonstrated that there was no evidence of ongoing rounds of viral replication due to a lack of detectable accumulation of diversity within the sequence data despite higher levels of immune activation within the gut [45]. This latter result was confirmed by Josefsson et al. [54] and, later, Simonetti et al. [77] who found minimal genetic changes over time and no evidence for compartmentalization between the periphery and the gut after long-term therapy.

Although there is some debate regarding the compartmentalization of HIV in lymphoid tissue, the central nervous system (CNS) is one such compartment in which heavy restriction of gene flow affects the population structure [9–11, 111]. The compartmentalization of the CNS has been found to be strongly associated with HIV-Associated Dementia (HAD) [112, 113]. Studies by Schnell et al. [9, 10] and later, Sturdevant et al. [11] found two distinct types of compartmentalization within the cerebrospinal fluid (CSF). The authors reported that the T cell tropic virus found in the CSF was generally clonal in nature, and associated with pleocytosis, whereas macrophage-tropic virus (CD4+ low) was generally diverse and contained variants not represented in the plasma [9, 10]. These results suggested that HIV could replicate in at least two cell types within the CNS, but the authors noted that there was no relationship between the tropism of the virus and HAD diagnosis [11]. A recent study by Stefic et al. [111] attempted to enumerate differential selective pressures between the blood and CNS in the context of neutralizing antibodies. The authors reported that variants in the CNS had no differential ability to escape autologous neutralization when compared to the blood, but that there was a general increase in resistance to broadly neutralizing antibodies that was independent of compartmentalization, suggesting that the CNS could have clinical implications for immunotherapies [111].

Multiple studies have shown that the genital and genitourinary tracts are another site of compartmentalization within an HIV-infected patient [114–116]. However, in contrast to these studies, Bull and colleagues published two studies showing that female genital tract sequences are typically monotypic in nature, most likely due to cellular clonal expansion of single variants [105, 117]. Bull and colleagues later showed that these monotypic populations do not form distinct lineages over time and are well mixed with the blood [118]. In addition, a study by Chaillon et al. [119] found evidence of compartmentalization between semen and blood, but that this structure did not persist over the timepoints analyzed. Taken together, these studies show that there is a complex interplay between the plasma and various anatomical sites throughout the body and that eradication strategies may require monitoring of both the blood and these anatomical sites.

### Production of virus from clonally-expanded populations of infected cells

When HIV infected cells proliferate, proviral sequences are replicated with the high-fidelity cellular DNA polymerase, resulting in identical copies of the original provirus. Evidence for clonal proliferation as the source of persistent viremia, rather than ongoing cycles of viral replication, was first provided by finding the persistence of a large proportion of identical plasma sequences during residual viremia [46, 53]. This suggested that the identical viruses found in plasma may be produced by cells that have undergone clonal proliferation. The large majority of virus producing clones have defective proviruses, as intact *gag* alone is required for non-infectious particles to assemble [120]. Defective proviruses are the likely major contributor to persisting low level viremia. This explains the large proportion of identical sequences in residual viremia and the lack of linkage of persisting low level viremia with replication competent virus or virus rebounding after therapy interruption [46, 100]. Recently, novel assays to investigate HIV integration sites have been developed, which revealed that proviral integration in or near growth genes is associated with selective survival and expansion of infected CD4+ T cell clones [16, 17]. As described previously, it has also been shown that CD4 clones could harbor intact and replication-competent proviruses [18, 20, 77, 81] and that these clones contain members that are transcriptionally active [77, 78] and can be the source of persistent viremia [77] and of viral rebound [86]. In addition, recent studies have focused on the different T cell subsets with respect to locating clones with intact proviruses. Lee and colleagues found that identical variants were preferentially in Th1-polarized cells [38] and Hiener et al. [39] found intact proviruses in effector memory T cells. Taken together, these studies emphasize the role of cellular proliferation in maintaining of the HIV reservoir and suggest that further studies are needed to determine the association between different cell subsets and the clonal expansion of infected cells. It has been further suggested that there is an inverse relationship between the size of proviral clones and their probability of harboring replication-competent virus [20]. This may be explained by CD4 clones with large internal proviral deletions being less susceptible to CTL killing [88]. Taken together this explains why residual viremia in patients on long term ART may predominantly originate from defective proviruses and why there is an absence of correlation of residual viremia and quantitative infectious virus recovery [121].

## Emergence of drug resistance

Although ART is highly effective at inhibiting viral replication, drug resistant variants can emerge if ART is taken intermittently or if resistance mutations were present in the population prior to its initiation. HIV drug resistance was first observed with zidovudine/azidothymidine (AZT) monotherapy with the selection of thymidine-associated mutations (TAMs) in the reverse transcriptase gene that were likely present at low levels prior to AZT exposure [122]. In contrast, triple combination ART, which first included either a protease inhibitor (PI) and two nucleos(t)ide reverse transcriptase inhibitors (NRTIs) or a non-nucleoside reverse transcriptase inhibitor with two NRTIs, resulted in sustained viral suppression in the majority of patients and a low prevalence of drug resistance in patients with high levels of adherence [123–125].

The remarkable success of combination ART has two main explanations. First, variants carrying multiple drug resistance mutations are unlikely to be present in the viral population prior to ART and, therefore, cannot be selected when adherence is sufficiently high enough to virtually block further ongoing cycles of viral replication. The much lower frequency of virologic failure due to drug resistance on combination ART is consistent with studies showing a lack of viral replication and evolution on therapy. Secondly, when combination therapy includes drugs with a high genetic barrier (requiring multiple mutations for resistance), such as the newer integrase strand transfer inhibitors (INSTIs), or when mutations have a high fitness cost, the probability of their existence and selection is even lower [126]. In particular, resistance to the new INSTI, dolutegravir (DTG), when used in combination ART appears to be exceedingly rare. This phenomena can be explained by its high genetic barrier and the high fitness cost of the drug resistant variants [127]. Consequently, dual treatment combinations of DTG with lamivudine or rilpivirine are currently being investigated in clinical trials [128, 129]. Nevertheless, when patients who are INSTI-experienced, have inadequate adherence or received DTG monotherapy, resistance has occurred [130–132]. Thus, even regimens with high genetic barriers could be compromised by pre-existing resistance, inadequate regimen formulations and insufficient adherence. In addition to high genetic barrier, the potency of particular drugs has been related to their ability to prevent new rounds of infection in single-cycle replication assays, referred to as the instantaneous inhibitory potential (IIP). Drugs with a high IIP may contribute to highly durable regimens by virtually halting viral replication and thereby preventing viral evolution [133, 134]. Taken together, high potency and high genetic barrier regimens have contributed to the prevention of antiviral escape and the success of combination ART to prevent disease progression.

Considering the effectiveness of modern ART, it begs the question why virologic failure due to drug resistance still occurs. A major predictor of regimen failure is significant pre-existing drug resistance resulting from previous drug exposure [35, 135, 136], transmitted drug resistance [137], or possibly, high viral population size [3, 138]. However, even without pre-existing resistance, inadequate adherence could create a favorable environment for the stochastic emergence and subsequent selection of resistant mutants. As the different components of combination regimens have different half-lives, breaks in therapy could effectively result in monotherapy of the component with the longest half-life, leading to the selection of drug resistance mutations. In particular, breaks in therapy containing NNRTIs that have long half-lives, are associated with a high risk of failure [139, 140].

## Conclusions

Studies on intra-patient HIV genetic diversity on ART have contributed to our understanding of the establishment and maintenance of the reservoir that results in viral rebound when ART is interrupted [16, 17, 46, 53, 77, 86]. To date, scientific consensus has established that HIV replication is virtually halted in the peripheral blood of individuals fully suppressed on ART as most studies conclude that the viral population in PBMC does not diverge due to viral replication from pre-therapy populations for up to about 20 years on potent and adherent therapy [40, 53, 54, 102, 103]. However, whether viral replication persists in tissues, such as lymph nodes and gut, to levels that can maintain the HIV reservoir is still controversial [44, 45, 90, 104, 107, 110]. Because newly infected cells are not detected in the peripheral blood even after many years on ART, if viral replication persists in tissues, it indicates that these cells rarely migrate outside of their anatomical site of infection. Studies on proviral compartmentalization aim to investigate viral gene flow to better understand the migration patterns of infected cells and address the question of ongoing HIV replication during ART in tissues. However, such studies, thus far, have come to contradicting conclusions with some showing evidence of compartmentalization between blood and lymphoid tissues [44, 107] and others showing a lack of compartmentalization [45, 54, 110]. The conflicting findings may be due to differences in methods used to obtain the sequence data and analyze them or in differences in the region or length of the gene fragments investigated. More in depth studies on HIV populations in multiple genes are needed to resolve this controversy and to determine if ongoing cycles of viral replication contribute to maintaining the HIV reservoir on ART.

It is now well established that a small fraction of the cells that were likely infected prior to starting ART or during treatment interruptions can persist on long-term ART through cellular proliferation. It is likely through silencing of viral gene transcription (latent infection) that these cells survive and divide despite infection. Furthermore, the proliferation of infected cells is, in some instances, is driven by the interruption of the cell cycle by integration of HIV proviruses into oncogenes or genes that regulate cell growth [16, 17]. In one case, it was demonstrated that a large HIV infected cell clone was the source of persistent viremia and carried an archived, intact provirus that was capable of producing infectious virus in in vitro experiments [77]. This study was followed by others demonstrating that clones of cells carrying intact and replication-competent proviruses is not uncommon in individuals on suppressive ART [18, 20, 81]. These studies clearly show that a common reservoir for HIV infection during ART is the persistence and proliferation of cells infected with intact proviruses. More studies are needed to determine if such variants are always archival or if they can emerge from new rounds of infection in tissues during ART and to understand the distribution of cell clones across different anatomical compartments. Furthermore, single-cell studies are needed to confirm if the mechanism that allows the persistence of such clones is, indeed, HIV latency. Understanding the mechanisms that maintain the HIV reservoir will guide the design of strategies to eradicate the infection, such as the further development of agents aimed at driving infected cells out of latency, without inducing further cellular proliferation, so that HIV proteins can be targeted by, perhaps, a boosted immune system. Future studies on HIV diversity and evolution will likely guide this process and may contribute to evaluating the efficacy of curative interventions for HIV infection.

## Abbreviations

ART: antiretroviral therapy
PBMC: peripheral blood mononuclear cells
LN: lymph node(s)
APOBEC: apolipoprotein B mRNA editing enzyme, catalytic polypeptide-like
CNS: central nervous system
CSF: cerebral spinal fluid
IIP: instantaneous inhibitory potential
454: 454 pyrosequencing
